# Supplementary material for: Experimental evidence of symmetry breaking of transition-path times
Source: Nat Commun. 2019 Jan 4;10:55. doi: 10.1038/s41467-018-07873-9 (PMC6320364; doi:10.1038/s41467-018-07873-9)
Supplement: Supplementary file 1 — Supplementary Information [file 41467_2018_7873_MOESM1_ESM.pdf]

Experimental evidence of symmetry breaking of transition-path times

Gladrow *et al*.

Supplementary Note 1: Calibration of holographic phase-gradient forces

As we describe in the Methods section, we calibrated the phase-gradients used in our first-passage times experiments in a self-consistent way with the probability of right, $P_{\rightarrow}$, and left exits $P_{\leftarrow}$. For the sake of readability, we repeat the expressions in Supplementary Equations 1 and 2.

$$P_{\rightarrow}(f) = \frac{1}{1+e^{\frac{fL}{2\,k_BT}}} \qquad (1)$$

$$P_{\leftarrow}(f) = 1 - P_{\rightarrow}(f) = \frac{e^{\frac{fL}{2\,k_BT}}}{1+e^{\frac{fL}{2\,k_BT}}} \qquad (2)$$

However, we do not control the forces f directly, but merely a phase-gradient parameter p (dimensionless), which controls the relative offset of the hologram in the x-axis from the centre. The aim of the calibration is therefore to find the relation $f(p)$.

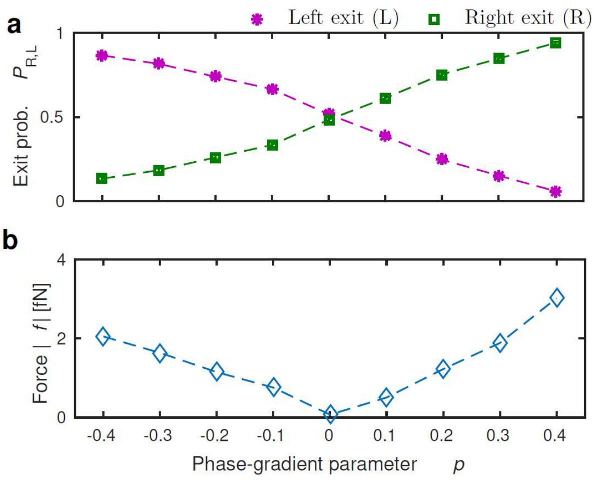

Supplementary Figure 1. Calibration of phase-gradient forces. **a** Exit probability for different phase-gradient parameters *p*. Error bars are smaller than the symbol size and represent the standard error of the mean. **b** Inferred dependence of magnitude of forces |*f*| on the phase-gradient parameter *p*.

We furthermore need to ensure that the diffusion coefficient does not depend on the line trap used to create force landscapes in our microfluidic experiments. As shown in Supplementary Figure 2**a**, the Diffusion coefficients stay within a narrow band of values around 0.13 $\mu m^2 s^{-1}$, but increase slightly near the entrance[1]. This shows, that the phase-gradients we use does not affect the roughness of the laser-induced energy potential and hence does not affect the diffusivity profile of the colloid.

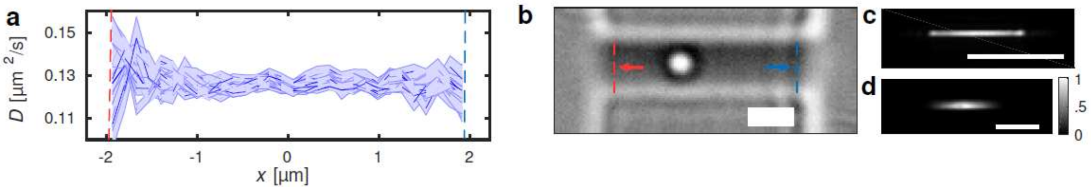

Supplementary Figure 2. Details of microfluidic experiments. **a** Diffusion coefficients for all phase-gradient parameters *p* used in Supplementary Figure 1. The dashed lines indicate the boundaries of the interval used in the exit time experiments (Fig. 2 in the main text). **b** Picture of microfluidic channel. The scale bar represents 1 µm. **c** Intensity graph of the line trap. Scale bar represents 8 µm. **d** Distribution of colloid localizations. The scale bar here represents 1 µm.

Supplementary Note 2: Exit time distribution of colloids in a pressure-driven hydrodynamic flow

The uphill and downhill exit-path-time symmetry described in the main text also holds for forces of different nature. We repeated the experiment described in Fig. 2 in the main text with pressure-drive flows. The laser is here only used to reset the colloids in the channel centre. The cumulative distribution of exit-path times is shown in Supplementary Figure 3 below.

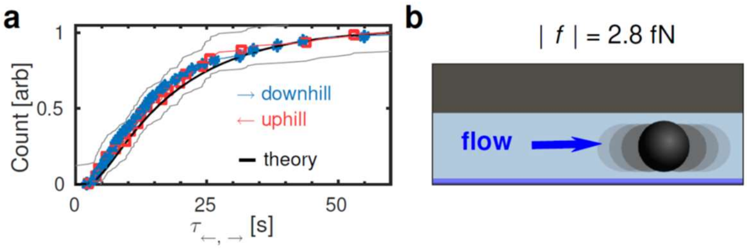

Supplementary Figure 3. Exit-path-time symmetry under pressure-driven flows. **a** Cumulative distribution of uphill (red) and downhill (blue) paths. The gray lines indicate the boundaries of the Kolmogorov-Smirnov test used to assert statistical significance of the comparison of distributions. **b** Sketch of the experiment.

Supplementary Note 3: Transition-path-time asymmetry cannot be used to estimate entropy production

In the main paper we show that an asymmetry in the distribution of transition-path times requires the system to be both out of equilibrium and multi-dimensional. Therefore, if asymmetric distributions for transition-path times are observed, this necessarily implies that both conditions are realized. One could ask whether, given the link between non-equilibrium dynamics and transition-path-time asymmetry, the latter could be used to characterize the non-equilibrium state, for example, by measuring entropy production. In this section, we show that this is not possible. If the system fulfils certain symmetry conditions (see Supplementary Figure 3), transition-path times will be symmetric in the presence of an arbitrarily large entropy production.

We consider again the hairpin example discussed in the main text in the results section, paragraph Breakdown of transition-path-time symmetry in DNA-hairpins. We consider transitions between the low-force folded state F_- to the high force unfolded state U_+ (forward transition) and vice versa (backward transition). In these transitions, the asymmetry of path times arises because transitions U_+ to F_- occur (preferentially) through one path (via F_+) while transitions from F_- to U_+ happen preferentially through U_- and the energy profiles along these two paths are different (which implies that one is faster than the other). As discussed below, under specific circumstances the force profile along the two paths (via F_+ or U_-) can become identical. In these cases, although forward or backward transitions still choose different paths (i.e. entropy production is finite), the transition-path times become experimentally indistinguishable.

In Supplementary Figure 3, we give an example of how this symmetry can arise in the hairpin system. The transitions between the folded and unfolded states of the hairpin are governed by its force dependent free-energy landscape. Folding, K_F, and unfolding, K_U, rates vary

exponentially with force f as can be seen from Supplementary Equations 3 and 4.

$$K_U(f) = K_0 e^{+X_U f/k_B T} \qquad (3)$$
$$K_F(f) = K_0 e^{(\Delta G_0 - X_F f)/k_B T} \qquad (4)$$

The extension of the unfolded and folded state is denoted by X_F and X_U respectively, while ΔG_0 denotes the difference in energy between the states in absence of external forces.

In the case of the hairpin used in our experiments, the different parameters take the values listed in Supplementary Table 1.

K_0	4x10[15] s[-1]
ΔG_0	60 k_BT
X_U	9.6 nm
X_F	8.1 nm

Supplementary Table 1. Numerical values of physical variables in our hairpin experiment.

As Supplementary Table 1 shows, the difference between X_U and X_F is relatively small. Consequently, it is possible to choose f_+ and f_- such that $K_U(f_+) \sim K_F(f_-)$ and $K_U(f_-) \sim K_F(f_+)$ (Supplementary Figure 3**a**). However, this is not always the case (see panel **b**). In general, this symmetry is not present (Supplementary Figure 3**b**). The symmetry is evident in the experimental trace, that shows the four states are roughly equally populated (panel **c**). In absence of this symmetry, the states are visibly not equally populated (see panel **d**).

In presence of the special symmetry discussed above, the transition-path time asymmetry is greatly reduced compared to the general case (Supplementary Figure 3**e**) and any path time asymmetry is hardly detectable, while it is evident in the more asymmetric case (Supplementary Figure 3**f**).

Although we have not quantified the entropy production in the driven hairpin system, we have shown that the asymmetry between transition times is, in cases of special symmetry, undetectable. This is the case despite strong driving, i.e. at finite entropy production. Therefore, transition-path-time asymmetry cannot be used to estimate entropy production. It might still be possible to use transition-path times to give a lower bound.

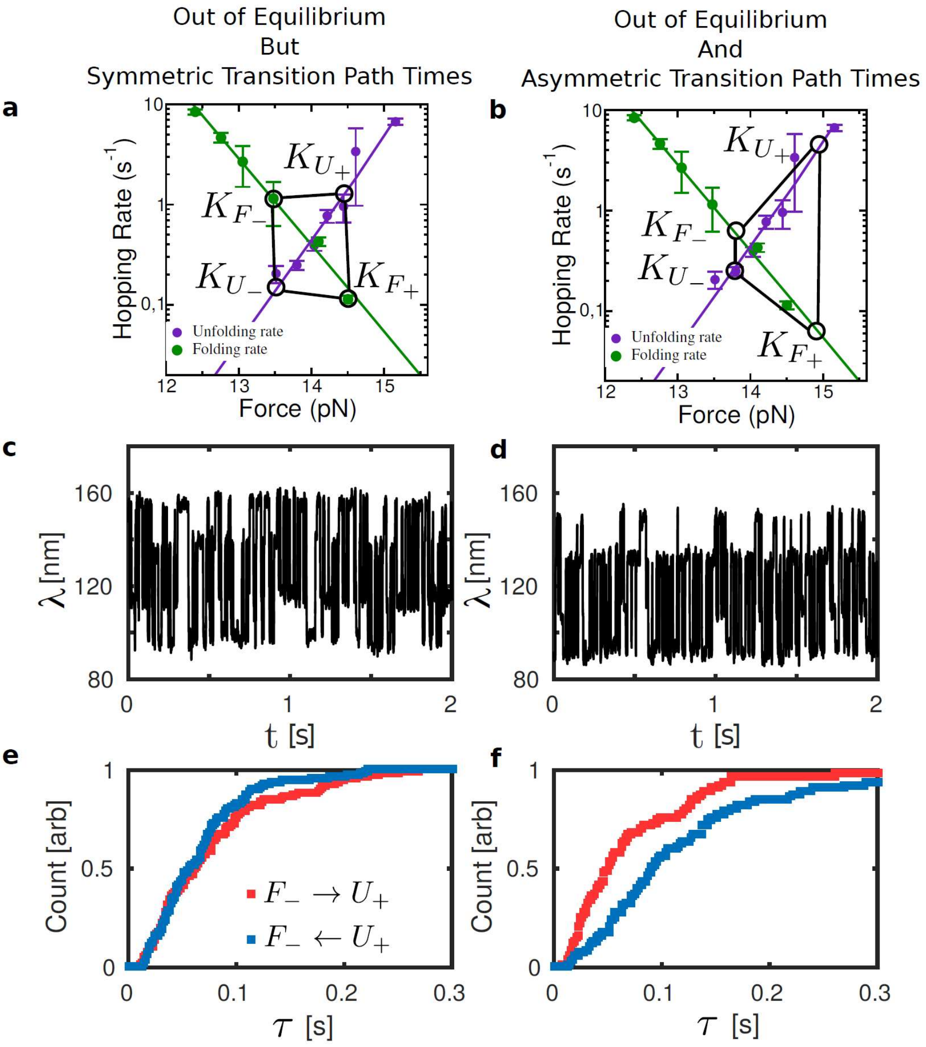

Supplementary Figure 4. DNA-folding and unfolding transition dynamics in and outside of equilibrium**. a** Folding (K_F) and unfolding (K_U) rates for the hairpin used in the experiments reported in the main text. The folding rate decreases with force while the unfolding rate increases with force. Folding and unfolding rates are equal at the coexistence force (f_c : $K_F(f_c) = K_U(f_c)$). Under telegraph noise, the force imposed on the hairpin switches between two values, f_+, f_-. When these two values are equally spaced around f_c , an approximate symmetry arises: $K_F(f_+) \approx K_U(f_-)$ and $K_U(f_+) \approx K_F(f_-)$. The error bars represent the standard error of the mean. **b** The symmetry of panel **a** is broken in the general case, where the rates differ. Error bars show the standard error of the mean. **c** Sample trace of DNA-hairpin elongation λ over time t (corresponds to panel **a**). **d** Similar to panel **c**, for forces that lead to different rates (corresponds to panel **b**). **e** DNA-hairpin experiments that fulfil the symmetry in panel **a**, the transition-path times for forward and backward transitions become experimentally indistinguishable. **f** Transition-path times are different in the general case (case shown in panel **b**).

Supplementary References

1. Dettmer, S.L., Pagliara, S., Misiunas, K. & Keyser, U.F. Anisotropic diffusion of spherical particles in closely confining microchannels. *Phys. Rev. E* **89**, 062305-062311 (2014).
